# Virtual histology of archaeological human deciduous prenatal enamel through synchrotron X-ray computed microtomography images

**DOI:** 10.1107/S160057752101208X

**Published:** 2022-01-01

**Authors:** Alessia Nava, Patrick Mahoney, Luca Bondioli, Alfredo Coppa, Emanuela Cristiani, Luciano Fattore, Gina McFarlane, Diego Dreossi, Lucia Mancini

**Affiliations:** a School of Anthropology and Conservation, University of Kent, Giles Lane, Canterbury CT2 7NZ, United Kingdom; b Department of Maxillo-Facial Sciences, Sapienza University of Rome, Via Caserta 6, Rome 00161, Italy; c Department of Cultural Heritage, University of Bologna, Via degli Ariani 1, Ravenna 48121, Italy; d Department of Environmental Biology, Sapienza University of Rome, P. le Aldo Moro 5, Rome 00185, Italy; e Department of Chemical Engineering Materials Environment, Sapienza University of Rome, via Eudossiana 18, Rome 00184, Italy; f Elettra Sincrotrone Trieste SCpA, SS 14 Area Science Park, Basovizza, Trieste 34149 Italy

**Keywords:** virtual dental histology, prenatal dental enamel, synchrotron X-ray microtomography, enamel daily secretion rates

## Abstract

Virtual histology is increasingly utilized to reconstruct the cell mechanisms underlying dental morphology for fragile fossils when physical thin sections are not permitted. Yet, the comparability of data derived from virtual and physical thin sections is rarely tested. Here, the results from archaeological human deciduous incisor physical sections are compared with virtual ones obtained by phase-contrast synchrotron radiation computed microtomography (SRµCT) of intact specimens using a multi-scale approach. Moreover, virtual prenatal daily enamel secretion rates are compared with those calculated from physical thin sections of the same tooth class from the same archaeological skeletal series. Results showed overall good visibility of the enamel microstructures in the virtual sections which are comparable to that of physical ones. The highest spatial resolution SRµCT setting (effective pixel size = 0.9 µm) produced daily secretion rates that matched those calculated from physical sections. Rates obtained using the lowest spatial resolution setup (effective pixel size = 2.0 µm) were higher than those obtained from physical sections. The results demonstrate that virtual histology can be applied to the investigated samples to obtain reliable and quantitative measurements of prenatal daily enamel secretion rates.

## Abstract 2

The implementation of synchrotron radiation X-ray microtomography for non-destructive visualization of the dental prenatal enamel microstructure of intact archaeological human deciduous teeth is presented and compared with the physical thin-sectioning of the same archaeological dental series. Prenatal daily enamel secretion rates, *i.e.* the distance between two adjacent daily enamel microstructures, have been measured with the two approaches. Results show a good agreement between physical and virtual histology.

## 1. Introduction

Human teeth hold a wealth of information about an individual’s growth, development, diet, chemistry and non-specific illnesses (Hillson, 2014 ▸; Guatelli-Steinberg, 2016 ▸). Because of this, histologists routinely draw on the cell mechanisms underlying tooth morphology to reconstruct enamel growth in order to contribute insights into the evolution of hominins, their phylogenies and life history (Dean, 2016 ▸). Relative to permanent teeth, human deciduous teeth have been less studied for this purpose but they retain unique information about prenatal ontogeny (Nava, Bondioli *et al.*, 2017 ▸; Nava, Coppa *et al.*, 2017 ▸; Mahoney, 2015 ▸), and aspects of maternal diet, health and mobility during pregnancy (Nava, Coppa *et al.*, 2017 ▸; Nava *et al.*, 2020 ▸; Li *et al.*, 2020 ▸).

Enamel forms incrementally with a 24 h circadian rhythm and retains a record of this growth in the form of incremental markings known as cross striations (Lacruz *et al.*, 2012 ▸; Zheng *et al.*, 2013 ▸). A fundamental dental-growth parameter is the amount of enamel deposited by cells over this 24 h period (*i.e.* daily secretion rate: DSR). Measurements of prism lengths between two adjacent cross striations is the amount of enamel deposited over 24 h. There are many applications of DSRs in studies of biological anthropology and human evolution. DSRs calculated from physical thin sections have been used to reconstruct the speed at which enamel cells form a human tooth crown in archaeological and contemporary series (Birch & Dean, 2014 ▸; Mahoney, 2012 ▸; Nava, Bondioli *et al.*, 2017 ▸), and have provided insight into the evolution of hominin dental development and life history through the analysis of fossil dental specimens (Nava *et al.*, 2020 ▸; Macchiarelli *et al.*, 2006 ▸; Dean *et al.*, 1993 ▸, 2001 ▸, 2010 ▸, 2016 ▸; Smith *et al.*, 2018 ▸; Austin *et al.*, 2013 ▸; Rosas *et al.*, 2017 ▸; Lacruz *et al.*, 2008 ▸; Dean & Smith, 2009 ▸; Joannes-Boyau *et al.*, 2019 ▸). Prenatal enamel usually shows less distinct microstructures with respect to the postnatal one, possibly as a consequence of the protected and buffered intrauterine environment in which the tissue develops (Nava, Bondioli *et al.*, 2017 ▸; Norén, 1983 ▸; Rythén *et al.*, 2008 ▸). For this reason, data on prenatal enamel DSRs are scarce and mostly available from modern reference collections of exfoliated/extracted deciduous teeth (Mahoney, 2012 ▸, 2015 ▸; Birch & Dean, 2014 ▸; Nava, Bondioli *et al.*, 2017 ▸; Dean *et al.*, 2020 ▸).

Most studies of enamel histology involve destructive thin sectioning of teeth (*e.g.* Mahoney, 2015 ▸; Nava *et al.*, 2019 ▸). Increasingly, anthropological studies have turned to non-destructive virtual histology of rare and fragile fossil specimens of mammalian teeth. Virtual histology is an advanced imaging technique for phenotyping, depiction and discrimination of various biological tissues (Albers *et al.*, 2018 ▸; Dullin *et al.*, 2017 ▸; Sanchez *et al.*, 2012 ▸; Tafforeau & Smith, 2008 ▸). This typically requires phase-contrast synchrotron radiation computed microtomography (SRµCT) (Le Cabec *et al.*, 2015 ▸, 2017 ▸; Smith *et al.*, 2007 ▸, 2015 ▸; Nava, Coppa *et al.*, 2017 ▸; Tafforeau *et al.*, 2006 ▸, 2007 ▸, 2012 ▸; Xing *et al.*, 2019 ▸; Tafforeau & Smith, 2008 ▸). Virtual histology can be considered non-destructive, as long as the experimental parameters are set to reduce the total dose delivered and therefore minimize the potential deleterious effects of X-rays on ancient DNA that could be preserved in teeth and made available for possible future analysis (Immel *et al.*, 2016 ▸).

The comparability of growth parameter estimates for the enamel fine microstructures (*i.e.* those whose dimensions are on the order of a few micrometres) derived from these two approaches is largely unexplored. One study conducted on permanent teeth using phase-contrast SRµCT – with a sub-micrometric pixel size – reported that enamel-growth parameters calculated from physical thin sections and virtual histology were comparable with a maximum difference of less than 1% (Tafforeau & Smith, 2008 ▸).

This study aims to analyze prenatal enamel from the Roman Imperial necropolis of Velia (I–II centuries CE, Salerno, Italy) (Fiammenghi, 2003 ▸) using 3D virtual histology of intact deciduous teeth. The goal is to extend the validation of virtual histology to deciduous teeth and particularly to the less readable prenatal enamel (Hillson, 2014 ▸). Here, we use the SRµCT setup of the SYRMEP beamline (Tromba *et al.*, 2010 ▸) of the Elettra synchrotron facility in Basovizza (Trieste, Italy). Virtual histology is conducted in propagation-based phase-contrast mode, which allows us to consistently image and quantify the fine growth markers in prenatal enamel. For the first time, we compare virtual data acquired using different effective pixel sizes to DSRs for the same tooth type and archaeological skeletal series (Nava, Bondioli *et al.*, 2017 ▸) calculated from physical thin sections.

## 2. Materials and methods

A sample of four intact deciduous central upper and five lower incisors (Table 1 ▸) were selected from an archaeological sample of infants from the large Imperial Roman necropolis at the ancient port of Velia (I–II centuries CE, Campania, Southern Italy) (Fiammenghi, 2003 ▸; Craig *et al.*, 2009 ▸; Bondioli *et al.*, 2016 ▸). This skeletal collection was chosen because previous histological analysis of the prenatal enamel of upper and lower central deciduous incisors revealed cross striations and other incremental markings were well preserved (Nava, Bondioli *et al.*, 2017 ▸).

The age-at-death of the individuals selected for the present analysis ranges between 2 and 12 months based on skeletal and dental development (AlQahtani *et al.*, 2010 ▸; Cunningham *et al.*, 2016 ▸; Ubelaker, 1989 ▸; Buikstra & Ubelaker, 1994 ▸). The skeletal collection is curated in the Museo delle Civiltà, Museo Nazionale Preistorico Etnografico Luigi Pigorini of Rome, to which one of the authors (LB) was affiliated and in charge of the collection at the time of the analysis.

### 2.1. Acquisition of the SRµCT dataset

SRµCT measurements were conducted at the SYRMEP beamline of Elettra using a multi-scale approach. At SYRMEP the X-ray beam is delivered by a bending magnet source and experiments were performed in a filtered white-beam configuration (filters: 1.5 mm Si, 2.0 mm Al) with a mean energy of ∼27 keV (estimated entrance dose of the order of kiloGray). At the time of measurement, the only microtomographic setup available for pink beam experiments was installed ∼17 m from the source. The mechanical limitation of this setup did not allow a tiling scanning procedure (Du *et al.*, 2018 ▸), so only a single scan was acquired for each tooth.

Projections were recorded with a macroscope camera based on a 16-bit, water-cooled sCMOS detector (2048 × 2048 pixels) coupled to a 15 µm-thick LSO:Tb scintillator screen. Using the variable optical zoom of the detecting system, effective pixel sizes of 2.0, 1.3 and 0.9 µm (see Table 1 ▸) were set corresponding to fields of view of about 4.1 × 4.1, 2.6 × 2.6 and 1.8 × 1.8 mm, respectively.

For each scan, we acquired 2000 projections over a 180° rotation with an exposure time/projection of 2.0 s (effective pixel size = 0.9 µm) or 1800 projections over a 180° rotation with an exposure time/projection of 1.5 s (effective pixel sizes of 2.0 and 1.3 µm).

The experiment was conducted in propagation-based phase-contrast mode with the sample-to-detector distance set to 150 mm for all measurements, independent of the pixel-size selection.

The slice reconstruction was carried out using the custom-developed SYRMEP Tomo Project (STP) software (Brun *et al.*, 2015 ▸).

Before reconstruction, a ring-removal algorithm (Rivers, 1998 ▸) was applied. Then, projections were processed by a single-distance phase-retrieval algorithm (Paganin *et al.*, 2002 ▸) with a δ/β parameter (ratio between the real and imaginary parts of the refraction index of the material *n* = 1 − δ − *i*β) chosen on an *ad hoc* basis for a good visualization of fine details in the tooth enamel balancing between contrast and spatial resolution. Since acquisition conditions were not ideal (that is to say homogeneous objects and a narrow X-ray energy bandwidth) a slight blurring can be observed in the reconstructed images. Paganin’s algorithm can be employed, with some caution, even on dense and multi-materials (Langer *et al.*, 2014 ▸; Arzilli *et al.*, 2015 ▸) and with polychromatic light (*e.g.* Myers *et al.*, 2007 ▸). In this case the δ/β ratio was set to 10.

### 2.2. Virtual histology extraction

The final reconstructed images for histological analyses were generated by importing the volumes in the *ImageJ* software (Schneider *et al.*, 2012 ▸), using the *Reslice* tool to generate a stack of images following the proper histological sectioning plane (buccolingual plane, passing through the tip of the dentine horn). The image stack was processed through the *ZProject average intensity* function varying the range of the stack to obtain different virtual thicknesses. In *ImageJ*, the brightness/contrast tool and the unsharp mask filter were used to enhance visualization of the features of interest, taking care to avoid the creation of artefacts.

### 2.3. Thin sectioning

The physical sectioning of the deciduous central incisors from the same collection was obtained using standard histological methods (Mahoney, 2015 ▸; Nava *et al.*, 2019 ▸). The teeth were first cleaned and embedded in ep­oxy resin (Epo-Thin, Buehler Ltd). A longitudinal buccolingual thin section passing through the centre of the cusp, ∼300 µm thick, was obtained using a diamond blade microtome (Leica 1600, Leica AG). The sectioned tooth was attached to a microscope slide, previously treated with liquid silane (3M RelyX ceramic primer), using a light-cured adhesive (3M Scotchbond multi-purpose adhesive) placed under UV light for 60 s. The sections were ground to a final thickness of ∼100 µm with water-resistant abrasive papers of different grits (Carbimet, Buehler Ltd), and then polished with a micro-tissue (Buehler Ltd) and alumina powder (MicroPolish II alumina 1.0 µm, Buehler). The enamel surface of the sections was etched using a phospho­ric acid gel (Etchant Delivery System, 3M Scotchbond) for 10 s, cleaned and finally mounted with a coverslip using Eukitt (mounting medium for microscopic preparations, O. Kindler GmbH & Co.).

Overlapping images of the entire crown were obtained with a microscope camera (Leica DFC 295, 6.55 × 4.92 mm CMOS sensor, 2048 ×1536 pixel imaging, 30 bits colour depth, with an effective pixel size of 0.52 µm when coupled with a 10× objective and 0.63× video optic adapter) paired with a transmitted light microscope (Laborlux S, Leica AG) using polarized light. The images were assembled into a mosaic using Microsoft *Image Composite Editor* [*ICE* 2.0 (https://www.microsoft.com/en-us/research/product/computational-photography-applications/image-composite-editor/)].

### 2.4. Daily enamel secretion rates

DSRs were collected from virtual and physical histological sections. Rates were calculated by measuring the length of prisms between five or six cross striations (representing four or five days of enamel secretion, respectively). This was repeated several times within the enamel regions where cross striations were visible so that a grand mean DSR could be calculated. Overall, a total of 112 DSRs were collected in the virtual thin sections and 51 in the physical thin sections. The Shapiro–Wilk normality test was used to check the normality of the DSR distributions. Non-parametric statistical tests were used to compare the DSR sample distributions because the Shapiro–Wilk test showed that the DSR distribution deviates from normality. The statistical package *R* (version 4.1.1) (R Core Team, 2021 ▸) was used for the statistical analysis and generation of graphs.

## 3. Results and discussion

### 3.1. Virtual histology: visualization of the enamel microstructures

**Figure 1:**
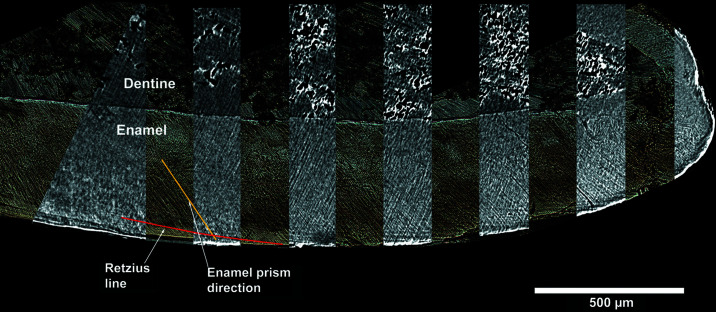
Velia 376 upper deciduous central incisor, superimposition of virtual histology reformatted slice portions on the classic histology image. Virtual histology: vestibular-lingual reformatted image from SRµCT, isotropic voxel size = 1.3 µm, slice thickness = 13 µm. The yellow line highlights the enamel prism direction, the red line highlights the Retzius line direction.

**Figure 2:**
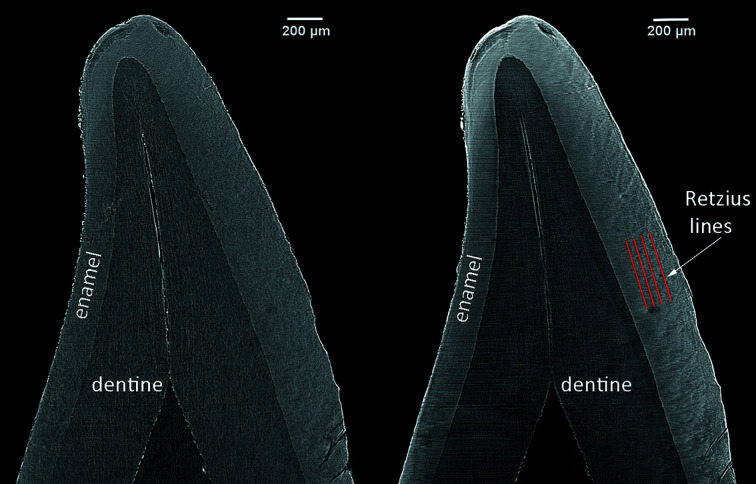
Velia 387 lower deciduous central incisor. Vestibular-lingual reformatted images from SRµCT, isotropic voxel size = 2.0 µm. Different slice thicknesses (left 2.0 µm, right 40 µm) of the same volume highlight different features. Horizontal lines visible in the images correspond to the plane of the tomographic slices.

A total of 18 synchrotron radiation microtomographic scans were acquired on 9 individual teeth from Velia (Table 1 ▸). Overall, the visibility of enamel microstructures in the reconstructed images was good and comparable to that of the physical sections (Fig. 1 ▸). Daily cross striations, a neonatal line and other accentuated markings are visible. The anatomical morphology of these features make them recognizable despite of the presence of linear artefacts (even more visible in Fig. 2 ▸ as horizontal stripes) related to a non-optimal ring removal procedure.

**Figure 3:**
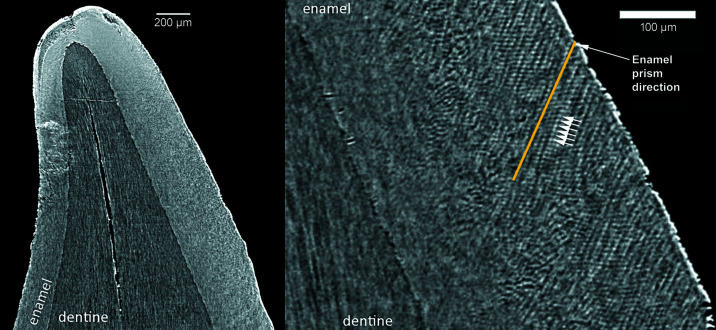
Velia 387 lower deciduous central incisor. Vestibular-lingual reformatted images from SRµCT, isotropic voxel size = 1.3 µm, slice thickness = 1.3 µm. The fine enamel microstructures are discernible. On the right, the magnification of a portion of the left image shows enamel prisms (yellow line) and cross striations (white arrows). Each dark-and-bright couple highlighted by the white arrows represents a single cross striation, *i.e.* 24 h of enamel deposition.

**Figure 4:**
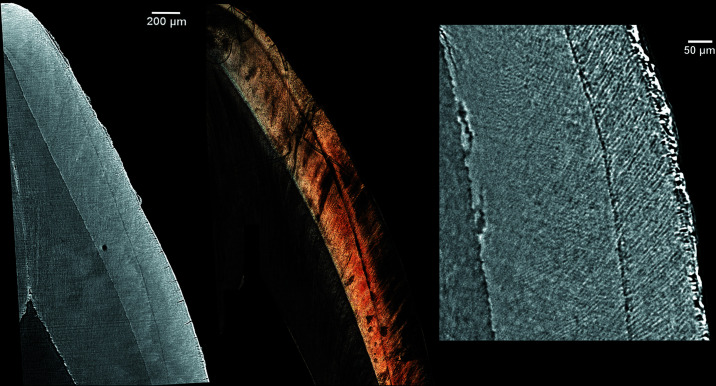
Velia 96 lower deciduous central incisor. Left: vestibular-lingual reformatted image from SRµCT, isotropic voxel size = 2.0 µm, slice thickness = 20 µm. Center: optical microscopy image of a vestibular-lingual histological section of the same tooth taken with 10× objective magnification (0.52 µm pixel size). Right: vestibular-lingual image from SRµCT, isotropic voxel size = 0.9 µm.

Retzius lines and the neonatal line were more visible on slices that are 20–130 µm thick (Fig. 2 ▸). Cross striations were more easily discernable in sections where the thickness of the reformatted image is equivalent to the voxel size (Fig. 3 ▸). The neonatal line was clearly visible in all the reconstructed and physical thin sections (Fig. 4 ▸).

### 3.2. Comparison of measurements at different voxel sizes of daily secretion rates

We were able to record DSRs in all images reconstructed with isotropic voxel sizes of 2.0, 1.3 and 0.9 µm (Fig. 3 ▸). DSRs were determined through multiple estimates for ten virtual histological sections across the entire prenatal portion of the crown.

**Figure 5:**
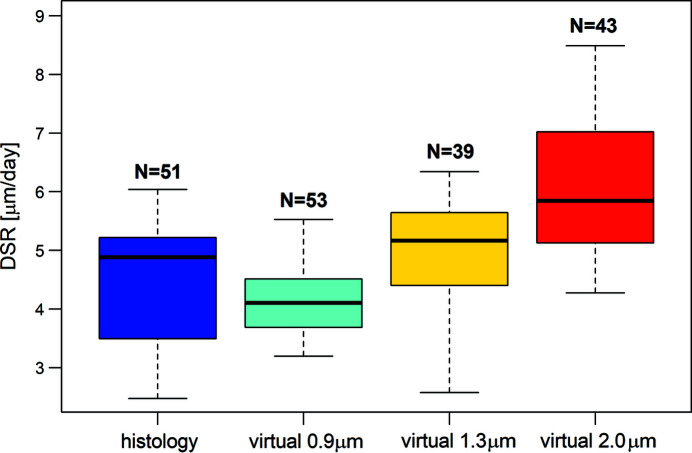
Box and whiskers plot of the DSR distributions in classical and virtual histology at different voxel sizes. Virtual 0.9: virtual histology at 0.9 µm pixel size. Virtual 1.3: virtual histology at 1.3 µm pixel size. Virtual 2.0: virtual histology at 2.0 µm pixel size. Bold line – median. *N* – number of samples.

Fig. 5 ▸ shows the boxplots comparing the distribution of DSR values in the virtual (isotropic voxel size of 0.9, 1.3 and 2.0 µm) and physical thin sections from deciduous central incisors of the same skeletal series (Table 2 ▸).

The Fligner–Killeen test for homogeneity of variance between real histology and different voxel size SRµCT measures shows a statistically significant difference between the DSR variances (chi-squared = 15.658, DOFs [degrees of freedom] = 3, *p* < 0.05). The Kruskal–Wallis test shows a statistically significant difference between the DSR distributions (chi-squared = 60.327, DOFs = 3, *p* < 0.01). The pairwise Wilcoxon rank-sum test with continuity correction and Bonferroni correction of probabilities shows no significant difference between the physical sections and SRµCT measurements at 0.9 or 1.3 µm voxel sizes. Significant differences were only detected between physical sections and SRµCT measurements at 2.0 µm voxel size (mean difference = 1.3 µm, *p* < 0.01). Measurements also differed significantly between the SRµCT measurements at 1.3 and 2.0 µm voxel sizes (mean difference = 0.97 µm, *p* < 0. 01) and slightly but significantly between 0.9 and 1.3 µm voxel sizes (mean difference = 0.66 µm, *p* < 0.05).

Overall, the virtual histological assessment of DSRs produces values comparable to those from the physical sections when the smallest voxel size is used. In fact, by increasing the voxel size, the partial volume effect (values of the greyscale levels associated with a single material changing in different parts of the image) is more evident. This artefact can partially mask features of interest if their size is within 2–3 times the voxel resolution and can cause an erroneous measurement of the parameters of interest (Bull *et al.*, 2013 ▸; Withers *et al.*, 2021 ▸), resulting in larger uncertainty of the DRS values (Table 2 ▸).

It is reasonable to compare the mean values between different individuals and between different measurement techniques. In fact, the DSR values obtained from the present virtual histological analysis are in agreement with those already estimated with classical histology for the same population and same tooth class, always in the prenatal enamel [Wilcoxon rank-sum test with continuity correction *W* = 3159.5, *p* > 0.05, reference data from the work by Nava, Bondioli *et al.* (2017 ▸)].

## 4. Conclusions

The results presented in this study show the comparison between the quantitative assessments of growth parameters (DSR) of dental deciduous enamel in histological thin sections through optical microscopy and SRµCT images acquired at the SYRMEP beamline of Elettra.

Several factors can affect spatial and contrast resolution in CT images, including X-ray beam energy and quality (geometry, monochromaticity, coherence), sample size and composition, the mechanical stability of the setup, detector performances (scintillator screen, optics, camera), image pre-processing (artefact removal, phase-retrieval), and reconstruction algorithm.

When using 1.3 and 0.9 µm pixel sizes for the phase-contrast SRµCT measurements, it was possible to produce data that varied only slightly from that obtained from physical thin sections. Indeed, the range of deciduous DSRs from the microtomographic setup lay within the range of DSRs known for modern human incisors (Mahoney, 2012 ▸), and those published previously for the same archaeological series analyzed here (Nava, Bondioli *et al.*, 2017 ▸).

Despite the above-mentioned factors affecting the reconstructed volumes and taking into account the quite heavy post-processing applied to these datasets, the deciduous enamel microstructures are well visible in the final images. Retzius lines and the neonatal line were easily identified with the microtomographic setup used. Even if images from physical thin sections tend to have higher contrast, virtual imaging has the obvious advantage that it allows extraction of a very high number of slices of different thicknesses

In summary, the results demonstrate that virtual histology can be applied to obtain reliable and quantitative measurements of prenatal DSRs in deciduous dental crowns.

## Figures and Tables

**Table 1:** Dental sample selected for SRµCT measurements U = upper dental arch, L = lower dental arch, i1 = central deciduous incisor; age at death estimated by morphological assessment. Li1 = lower central deciduous incisor, Ui1 = upper central deciduous incisor.

ID	Tooth type	Age at death	Pixel size (µm)
Velia 376	Li1	4–6 months	2.0
Velia 376	Li1	4–6 months	1.3
Velia 376	Ui1	4–6 months	2.0
Velia 376	Ui1	4–6 months	1.3
Velia 387	Li1	3–6 months	2.0
Velia 387	Li1	3–6 months	1.3
Velia 387	Ui1	3–6 months	2.0
Velia 387	Ui1	3–6 months	1.3
Velia 395	Li1	9–12 months	2.0
Velia 395	Li1	9–12 months	1.3
Velia 395	Ui1	9–12 months	2.0
Velia 395	Ui1	9–12 months	1.3
Velia 398	Li1	6–8 months	2.0
Velia 398	Li1	6–8 months	1.3
Velia 398	Ui1	6–8 months	2.0
Velia 398	Ui1	6–8 months	1.3
Velia 96	Li1	2–3 months	2.0
Velia 96	Li1	2–3 months	1.3
Velia 96	Li1	2–3 months	0.9

**Table 2:** DSR statistics for the dataset; SD = standard deviation

	Histology	Virtual 0.9 µm	Virtual 1.3 µm	Virtual 2.0 µm
Median	4.88	4.11	5.17	5.54
Mean	4.49	4.16	4.82	5.79
SD	1.01	0. 58	1.09	1.13
*n*	51	53	39	63
Min.	2.47	3.20	2.57	4.28
Max.	6.04	5.53	6.34	8.49
